# A Summary of the Anatomy and Current Regional Anesthesia Practices for Postoperative Pain Management in Total Knee Arthroplasty

**DOI:** 10.7759/cureus.2755

**Published:** 2018-06-07

**Authors:** Promil Kukreja, Joel Feinstein, Hari K Kalagara, Samuel R Huntley, Sung R Lee, Sameer Naranje, Ashish Shah

**Affiliations:** 1 Department of Anesthesiology & Perioperative Medicine, University of Alabama at Birmingham, Birmingham, USA; 2 Department of Orthopaedic Surgery, University of Alabama at Birmingham, Birmingham, USA

**Keywords:** multimodal analgesia, postoperative, pain management, regional anesthesia, nerve blocks

## Abstract

The planning and implementation of an effective postoperative pain management program depend on the surgical technique for total knee arthroplasty (TKA), the type of regional anesthesia, and the multimodal analgesia regimen. It is imperative to understand the surgical anatomy of TKA and the relevant nerve supply of the knee for optimum perioperative patient satisfaction with respect to pain management in the patient undergoing TKA. The commonly used regional techniques have their own specific benefits and limitations. The ideal postoperative pain management should be customized for a patient to achieve the goals of effective pain control, early ambulation, faster recovery, and discharge.

## Introduction and background

Total knee arthroplasty (TKA) is one of the most commonly performed procedures in orthopedic surgery. Although it is often used in the treatment of osteoarthritis of the knee, other etiologies, such as post-traumatic arthritis and inflammatory arthritis, are not uncommon. The demand for TKA is expected to increase 673% to 3.5 million procedures annually by 2030, and when including revision TKA procedures, an over eight-fold increase is likely [1]. The early postoperative period following a TKA is often associated with moderate to severe postoperative pain, and over a third of patients experience severe movement-related pain on the first and second postoperative days [2]. Although TKA has been established as an effective treatment for end-stage osteoarthritis, current postoperative pain management may be insufficient, resulting in delayed ambulation, prolonged physical therapy, and longer hospitalizations. This delay may translate to a higher complication rate (i.e. deep vein thrombosis and hospital-acquired infections), delayed discharge, increased hospital cost, and poor patient satisfaction [3-4]. There is a great emphasis on short hospitalizations, reduced postoperative complications, and faster recovery. The perioperative anesthesia for outpatient joint arthroplasty is essential, and its role will continue to evolve.

Numerous techniques, including a combination of neuraxial, high-volume, local infiltration of the surgical site with anesthetic cocktails and peripheral nerve blocks can be utilized in conjunction with multimodal analgesia to control postoperative pain, reduce opioid consumption, and facilitate faster functional recovery [5]. Femoral nerve block (FNB) is a well-established regional technique, which many consider as the standard of care for postoperative pain control [6]. The FNB fails to provide complete analgesia and as many as 60%-80% of patients with functioning FNB will complain of a clinically significant knee pain [7]. Sciatic nerve blocks (SNB) are used in conjunction with FNB to provide enhanced analgesia in patients undergoing TKA [8]. Even though the combination of SNB and FNB is a common practice in some centers, SNB remains among the least-performed peripheral nerve block by anesthesiologists, likely due to the difficulty in its performance in the subgluteal region, the risk associated with nerve injury, and an increased risk of patient falls due to foot drop [9]. The decision to perform a particular peripheral nerve block is based on risks and benefits supported by clinical evidence. A relatively new technique, adductor canal block (ACB), is commonly used as an effective alternative to FNB [10]. Many centers are combining these regional techniques with intraoperative periarticular injections performed at the time of surgical closure [11].

## Review

Anatomy and innervation of knee joint

Knee arthroplasty may entail either total or partial knee arthroplasty based on the extent of cartilage loss and knee compartments involved. The TKA is generally accomplished through a medial parapatellar approach with a midline longitudinal skin incision over the patella. Although no general consensus has been reached on the best surgical approach, preserving quadriceps tendon integrity may allow patients to have an earlier recovery of their extensor mechanism. Densely innervated areas include the posterior capsule, extensor retinaculum, and infrapatellar fat pad region.

The anatomic literature has revealed a marked variability in the distribution of nerve supply to the knee joint. An anatomical study of the nerve supply of the human knee was done by Gardiner to establish the consistency of certain branches and the definitive pattern of knee innervation [12]. Two consistently distinct groups of afferent nerves, such as the anterior group and the posterior group, are identified. The anterior group includes the afferent nerves of the femoral, saphenous, and common peroneal nerves. The posterior group includes the posterior articular and obturator nerves [13].

The afferent nerves of the anterior group predominantly supply the anteromedial and anterolateral capsules and the associated ligaments. Although articular cartilage is the primary site of injury in osteoarthritis of the knee, the cartilage is devoid of innervation and, therefore, no pain is transmitted from the tissue. As the articular cartilage wears away, the highly innervated periosteum becomes damaged and results in the joint pain. Other potential sources of knee pain include the subchondral bone, capsule, synovium, retinacula, and periarticular ligaments.

The terminal portions of the femoral nerve supplying the quadriceps muscle give rise to three articular afferents. The largest of these is the articular branch of the nerve to the vastus medialis that bifurcates beneath the medial retinaculum to supply a wide segment of the anteromedial capsule. The terminal branch of the vastus lateralis supplies the superolateral capsule. The terminal branch of the nerve to the vastus medialis supplies the medial articular structures, medial retinaculum and medial patella.

The saphenous nerve, itself the terminal branch of the femoral nerve, runs in the Adductor or Hunter’s canal anteromedial to the femoral artery. The infrapatellar branch of the saphenous nerve splits between the tendons of the sartorius and gracilis to innervate the inferomedial capsule, the medial collateral ligament (MCL), the patellar tendon, and the skin overlying the anterior aspect of the knee. Occasionally, the anterior division of the obturator nerve innervates part of the skin overlying the medial aspect of the knee.

The common peroneal nerve gives origin to two articular nerves, the lateral articular and recurrent peroneal nerves. The lateral articular nerve traverses near the fibular head to supply the inferior portion of the lateral capsule and the collateral ligament. The recurrent peroneal nerve divides around the fibular head to supply the anterolateral joint and the lateral collateral ligament (LCL).

The afferent nerves of the posterior group predominantly supply the posterior capsule and cruciate ligaments. The most consistent and largest nerve supplying the knee is the posterior articular branch of the posterior tibial nerve. It forms a popliteal plexus of nerve divisions deep in the popliteal fossa to supply the posterior capsule, the external part of the menisci and the anterior and posterior cruciate ligaments. The nerves from the plexus may reach as far anterior as the infrapatellar fat pad. The posterior division of the obturator nerve makes contributions to the popliteal plexus and supplies the posterior articular surface. The genicular nerves arise posteriorly from the tibial and common fibular nerves to form a network with the femoral and obturator nerves to supply the four corners of the knee joint.

**Figure 1:**
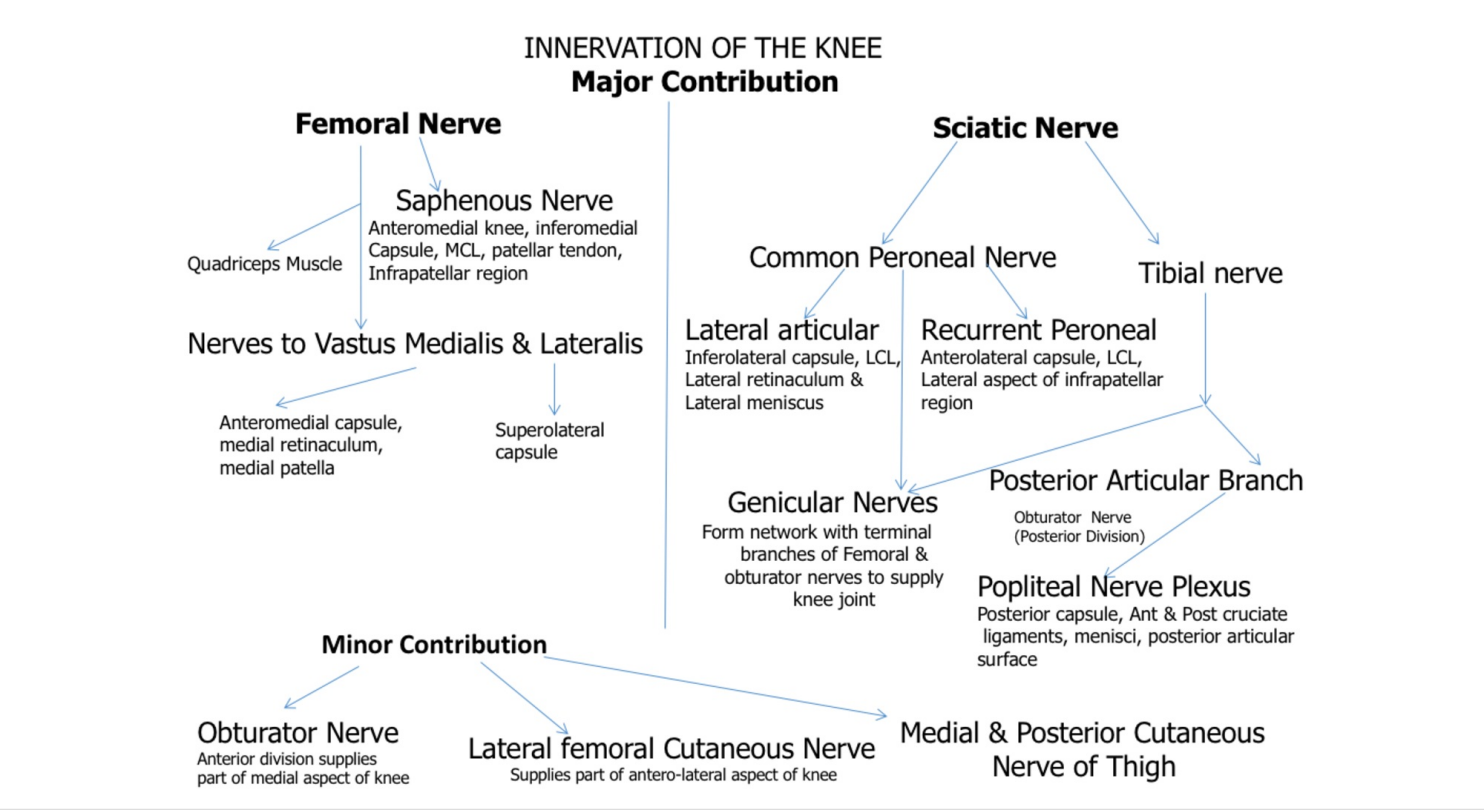
Major Nervous Contributions of the Knee

A summary of knee innervation is illustrated in Figure 1.

Discussion

The clinical decision of a particular regional anesthesia technique should be based on the knowledge gained from the surgical anatomy and innervation of the knee. Each peripheral nerve block used for the postoperative management of TKA has its own advantages and limitations.

The femoral nerve block (FNB) considered by many as the mainstay for effective pain control after TKA has some significant limitations. Continuous FNB is associated with a 1.0% to 2.5% incidence of prolonged quadriceps muscle weakness, nerve damage, and local infection. While the risk of cellulitis or systemic infection is very low, the incidence of bacterial colonization at 48 hours is 57% in patients with an FNB catheter [14]. The FNB is followed by significant quadriceps weakness, leading to delayed mobilization, and it also depends on the concentration of the local anesthetic used for the postoperative analgesia [15]. Furthermore, the FNB has been implicated with the risk of falls [16]. The other major limitation of FNB is that it does not control posterior pain originating in sciatic nerve territory [8].

The sciatic nerve block (SNB) can be used in conjunction with FNB to provide coverage in the posterior aspect of the knee. Some studies have shown the early analgesic benefit of SNB and decreased opioid consumption during the first 24 hours [17]. Although continuous SNB along with FNB has shown a significant improvement in analgesia beyond 24 hours [18], the review of literature found insufficient evidence to qualitatively define the effect of adding SNB to FNB for analgesia after TKA, even in the setting of continuous SNB [19]. The use of SNB renders some serious concerns for surgeons, including a difficulty in diagnosing peroneal nerve injury secondary to surgical injury. The SNB is also associated with lower extremity weakness, like foot drop leading to delayed ambulation and an inability to participate in physical therapy beyond that of the isolated FNB.

The adductor canal block (ACB) is a relatively motor (quadriceps)-sparing regional technique and used as an alternative to the FNB. Similarly to the FNB, an ACB can be a single dose or a continuous infusion of local anesthetic. The mid-thigh approach of the adductor canal blocks the largest sensory branch of the saphenous nerve, medial cutaneous femoral nerve, nerve to vastus medialis, and, possibly, the articular branches of the obturator nerve. The ACB targets mainly the sensory nerves with the exception of the nerve to the vastus medialis. By virtue of the distal anatomical location of ACB, the ACB provides a clinically significant preservation of quadriceps muscle strength as compared to the FNB while providing similar analgesia [20]. A few case reports have shown that the ACB can result in significant quadriceps muscle weakness [21]. The ACB has become widely popular given its quadriceps-sparing analgesia, but it does not provide a complete block of the knee [22]. In a recent randomized trial of 100 subjects undergoing anterior cruciate ligament (ACL) reconstruction, ACB preserves the quadriceps strength and provides non-inferior postoperative analgesia when compared with FNB [23]. Periarticular infiltration (PAI) of the knee relies on the knowledge of intraarticular neuroanatomy of the knee joint. The technique has been shown to reduce narcotic consumption and lower pain scores as compared with the use of the FNB [24]. Patients who received PAI demonstrated an increased capacity to perform a straight leg raise, better active knee extension, and an increased ability to ambulate than patients with FNB [25]. The PAI has the potential benefit of allowing increased muscle control with rehabilitation while avoiding complications associated with opiates and peripheral nerve blocks. The results of the PAI are inconsistent secondary to the unreliable spread of local anesthetics secondary to anatomical barriers and the infiltration technique utilized [11]. PAI is also limited by the duration of local anesthetics and an inability to titrate an effect with a catheter. PAI cannot be done in partial knee arthroplasty due to intact cartilage and the chondrotoxic effects of some local anesthetics and adjuncts on the cartilage used in the infiltration regimens. Genicular nerve blocks and genicular nerve radiofrequency ablation (RFA) techniques are gaining more popularity nowadays and are being used preoperatively for the control of postoperative TKA pain.

The duration of analgesia using the FNB is prolonged with the placement of a perineural catheter and the continuous infusion of local anesthetic, preferably with low concentrations and without bolus options for the infusion regimen. The continuous nerve block involves the inconvenience of carrying the catheter and local anesthetic reservoir/pump, infusion management, catheter site care, the cost and maintenance of equipment, the risk of infection, leakage, and accidental dislocation. Recently, the liposomal form of a local anesthetic (bupivacaine) has been introduced to provide prolonged postsurgical analgesia. Liposomal bupivacaine (Exparel, Pacira Pharmaceuticals, Inc., NJ, USA) is a multivesicular formulation of bupivacaine approved for administration into the surgical site but not for peripheral nerve blocks. A recent multicenter, randomized, double-blind study designed to meet the U.S. Food and Drug Administration (FDA) standard has shown that the FNB with liposomal bupivacaine (266 mg) resulted in modestly lower pain scores and reduced opioid use in the first 72 hours after TKA compared with the placebo, with an adverse event profile similar to the placebo [26]. While the results of this study showed that a single administration of liposomal bupivacaine in FNB could provide analgesia that lasts at least two days, more studies are clearly warranted to better define the potential role of liposomal bupivacaine in peripheral nerve blocks.

The liposomal bupivacaine (Exparel) is also used for PAI in TKA surgeries for postoperative pain control. The published study for unilateral knee replacement patients demonstrated a benefit in the Exparel group for length of stay and ambulation [27]. The study also concluded that the liposomal bupivacaine was non-inferior to FNB alone for combined pain scores throughout the patient’s hospital stay. The two available studies for TKA showed either equivalency or inferiority when the FDA-approved 266 mg dosing of Exparel was compared with less expensive traditional PAI [28-29]. Further studies are warranted to establish the utility of liposomal bupivacaine as a part of multimodal pain management protocol.

The posterior knee pain in the sciatic distribution is not covered by either the FNB or the ACB. There is some evidence of the spread of local anesthetic into the popliteal region when high volumes are used for ACB. The classical SNB can cause leg weakness, which interferes with ambulation. A selective motor sparing single injection, the sensory posterior articular nerves of the knee (SPANK) block has been introduced to cover the terminal nerve supply of the contiguous posterior knee capsule [30]. Although this new technique warrants further validation, it could potentially be used in conjunction with the continuous FNB or ACB. The basis of this technique may simplify the periarticular infiltration technique and used as a “rescue block” postoperatively for patients complaining of posterior knee pain [31]. In theory, the combination of the FNB or ACB with either periarticular infiltration or a selective sensory block of the articular nerves or genicular nerves could result in effective pain control with minimal muscle weakness. Although some pilot studies support these combination techniques, larger randomized trials are warranted to validate the results.

Another approach to provide effective analgesia for posterior knee pain is an infiltration of the interspace between the popliteal artery and the capsule of the posterior knee (iPACK). This iPACK technique can be used in combination with an adductor canal catheter to provide a balance of effective postoperative analgesia and the preservation of motor function, facilitating early ambulation and, ultimately, shortening the length of hospital stay following TKA [32].

Table 1 summarizes the utility, strengths, and weaknesses of the previously discussed anesthesia modalities.

**Table 1:** Comparison of Regional Techniques for TKA FNB = Femoral nerve block ACB = Adductor canal block SNB = Sciatic nerve block iPACK = interspace between the popliteal artery and the capsule of the posterior knee PAI = Periarticular infiltration MCL: Medial collateral ligament LCL: Lateral collateral ligament LA: Local anesthesia TKA = Total knee arthroplasty

Regional Technique	Target Regions of Knee	Benefits	Limitations
Femoral Nerve Block (FNB)	Anteromedial, inferomedial, and superolateral capsules, medial & inferior patella, MCL	Consistent coverage and duration, effectively used as a continuous block with a catheter, easy technique	Quadriceps weakness, fall risk, delayed ambulation, do not control posterior knee pain
Adductor Canal Block (ACB)	Anteromedial and inferomedial knee, MCL, partial patellar region	Minimal or no quadriceps weakness, early ambulation, and recovery	Do not cover anterior knee region completely, do not control posterior knee pain, inconsistent block results
Sciatic Nerve Block (SNB) (used in conjunction with FNB or ACB)	Anterolateral, inferolateral, and posterior capsules, anterior and posterior cruciate ligaments, LCL, Posterior articular region & menisci	Consistent coverage of the posterior knee, effectively used as a continuous block with a catheter	Leg weakness, delays ambulation, foot drop (rare), delays diagnosis of iatrogenic surgical nerve injury (rare)
iPACK	Terminal branches of genicular nerves and popliteal nerve plexus supplying the posterior knee region	Minimal or no motor weakness, covers posterior knee pain	Results vary based on the provider’s technique, anatomical barriers, and the surgical technique, effective when used in combination with either FNB or ACB
Periarticular Infiltration (PAI)	Terminal branches of genicular nerves and popliteal nerve plexus supplying the posterior knee region	Mostly done intraoperatively by surgeons under direct visualization.	High failure rates, technique dependent, inconsistent duration, & spread of LA

## Conclusions

The ideal regional technique for postoperative pain control after TKA should provide effective control of anterior and posterior knee pain with minimal muscle weakness to augment early functional recovery. The commonly used regional blocks target specific areas of the knee and have their own unique benefits with some specific limitations for each of them. There is no isolated regional block that can solely be utilized for complete TKA pain control. The varied analgesic approaches and institutional practices with regional techniques add challenge to standardize the pain management plan for TKA. The most effective practice includes incorporating a combination of motor sparing regional techniques along with a multimodal analgesic regimen with acetaminophen, nonsteroidal anti-inflammatory drugs (NSAIDs), and gabapentinoids that can provide adequate analgesia and facilitate early mobilization. There is little evidence supporting ACB and iPACK as compared to a combination of FNB and SNB, but more studies are warranted to establish this support. Standardized pain management strategies according to the institutional acute pain services is the key to success. It is imperative to select a combination of regional blocks or PAI to maximize the benefits and minimize the adverse effects.
